# Work Time and 11-Year Progression of Carotid Atherosclerosis in Middle-Aged Finnish Men

**Published:** 2008-12-15

**Authors:** Niklas Krause, Richard J. Brand, Candice C. Wong, Jussi Kauhanen, George A. Kaplan, S. Leonard Syme, Jukka T. Salonen

**Affiliations:** University of California, San Francisco, UC Berkeley Richmond Field Station; University of California, San Francisco, California; University of California, San Francisco, California; University of Kuopio, Kuopio, Finland; University of Michigan, Ann Arbor, Michigan; University of California, Berkeley, California; Oy Jurilab Ltd, Kuopio, Finland

## Abstract

**Introduction:**

Studies of the relationship between work time and health have been inconclusive. Consequently, we sought to examine the effect of work time on progression of atherosclerosis.

**Methods:**

This prospective study of 621 middle-aged Finnish men evaluated effects of baseline and repeat measures of work time on 11-year progression of ultrasonographically assessed carotid intima-media thickness (IMT) and interactions with cardiovascular disease. Multiple linear regression models adjusted for 21 biological, behavioral, and psychosocial risk factors.

**Results:**

Working 3 (minimum), 5 (medium), or 7 (maximum) days per week at baseline was associated with 23%, 31%, and 40% 11-year increases in IMT, respectively. The relative change ratio (RCR) at maximum vs minimum was 1.14 for baseline days worked per week and 1.10 for hours worked per year of follow-up. Significant interactions existed between cardiovascular disease and work time. Men with ischemic heart disease (IHD) who worked the maximum of 14.5 hours per day experienced a 69% increase in IMT compared with a 29% increase in men without IHD. The RCR ratio for IHD (RCR_IHD_/RCR_no IHD_) was 1.44 for hours per day. Similarly, the RCR ratio for baseline carotid artery stenosis was 1.29 for hours per day and 1.22 for hours per year.

**Conclusion:**

Increases in work time are positively associated with progression of carotid atherosclerosis in middle-aged men, especially in those with preexisting cardiovascular disease. Our findings are consistent with the hemodynamic theory of atherosclerosis.

## Introduction

Long work hours are associated with work accidents (1,2), reduced performance (3,4), retirement due to disability (5), high ambulatory blood pressure (6), acute myocardial infarction (7), hypertension (8), diabetes (9), and death from coronary heart disease (10). However, some studies have reported conflicting results (11-14), and reviews have characterized the relationship between work time and cardiovascular disease (CVD) as inconclusive (15,16). One reviewer notes that confounding, scarcity of prospective studies, and lack of repeat exposure measures in longitudinal studies may be partly responsible for inconsistent findings (15). The literature on employment status and health has been similarly inconclusive (17,18).

The primary aim of this study was to investigate the effect of work time on the progression of atherosclerosis while addressing methodologic issues by using a prospective design, using repeat measurements of work time, and adjusting for a comprehensive set of confounders. We addressed selection bias (the so-called healthy worker effect) by using a subclinical outcome measure, change in carotid artery intima-media thickness (IMT), instead of symptomatic CVD or death. A secondary aim of this study was to investigate whether occupational risk factors are more strongly associated with change in IMT among men with preexisting ischemic heart disease (IHD) or carotid artery stenosis (CAS) than among men without these conditions.

## Methods

### Study sample

Participants were part of an age-stratified, random, population-based sample of the prospective Kuopio Ischemic Heart Disease Risk Factor Study, which enrolled 1,516 Finnish men aged 42, 48, 54, or 60 years in August 1986. Ultrasound measurements of IMT of both common carotid arteries were conducted beginning in February 1987 on 1,229 participants. These men were invited to participate in a follow-up assessment approximately 4 years later. By that time, 47 had died or were suffering severe illness, 37 had moved or could not be contacted, and 107 refused, leaving 1,038 participants. Of these, 1,007 men were alive before the start of follow-up examinations scheduled from March 1998 through February 2001. An additional 58 men died before follow-up, 38 had a severe illness, 27 had moved or could not be contacted, 25 refused, and 5 did not participate for other reasons, which left 854 participants in the 11-year follow-up. Of these, 223 were excluded because they had not worked at all during the 11 years, 2 because they did not have an ultrasound examination at follow-up, 2 because of unreliable information on work time, and 6 because of missing values on 1 or more of the exposure variables, leaving 621 men for analyses. Missing values for 1 or more covariates were replaced by sample mean values in 11 (<1.8%) observations. Follow-up time between ultrasound examinations ranged from 9.23 to 13.82 years (mean, 11.13 years). The Committee on Human Research of the University of California, San Francisco, approved this study.

### Assessment of atherosclerotic progression

Ultrasound measurement of IMT in the carotid arteries is reliable, relates to the extent of disease in the coronary arteries, and has predictive validity with regard to risk of coronary events (19-26). Measurements of IMT were taken at approximately 100 sites along a 1.0- to 1.5-cm section of both the left and right common carotid arteries below the carotid bulb by using high-resolution B mode ultrasonography. Measurements were made while participants were supine, and the image focused on the posterior (far) wall. Additional technical details are published elsewhere (21). IMT was measured as the distance from the leading edge of the first echogenic line to the leading edge of the second echogenic line. Maximum IMT for the participant was defined as the average of the maximum IMT values from the right and left common carotid arteries. The maximum narrowing of the lumen is most relevant for arterial flow changes according to the hemodynamic theory. Our outcome measure was defined as the natural log of maximum IMT at 11 years minus the natural log of maximum IMT at baseline.

### Assessment of work time

An occupational physical activity interview was administered by trained interviewers at baseline, 4 years, and 11 years to men who had worked at least some time in the previous 12 months. Participants were asked when they arrived at work and when they left, how much break time they took, and how long they performed the following activities at work: sitting, standing, walking on level ground, walking on uneven ground, climbing stairs, or any other activities. The 12-month test-retest intraclass correlations for the metabolic units of occupational activity per typical workday was .69, indicating good reliability of the instrument and relative stability of exposure over time (27). Lifetime job stability among people living in the Kuopio region is high (28), reducing the probability of misclassification of work activities between follow-up examinations.

A self-administered questionnaire, completed at baseline, 4 years, and 11 years, provided information on occupation and work status. Participants who were not currently working were asked about the year when unemployment or retirement began and the number of days per week and hours per day worked in the previous job. Those who were working were asked number of days worked per week, hours and minutes worked per day, and days they missed work because of illness during the past 12 months.

The questionnaire and interview data were linked to the pension registers of the social insurance institution and the central pension security institute of Finland, which covers all old-age, disability, and early-retirement pensions of participants from baseline through the end of May 2000. These administrative data provided more exact retirement dates (month and year rather than just year) for the men who reported they had retired between follow-up surveys.

We used 5 measures of work time in our analyses: 1) days worked per week at baseline, 2) hours worked per day at baseline, 3) hours worked per week at baseline, 4) employment intensity during follow-up (years worked during follow-up divided by years of follow-up), and 5) average hours worked per year of employment during follow-up (average time worked per year of employment, accounting for hours worked per week, vacation time, and employment duration). In contrast to the previous measure, this measure accounts for varying hours worked per week assessed by repeat measures at 4 and 11 years

Covariates (Table 1) were assessed at baseline, 4 years, and 11 years. Details of the measurement of these variables have been described previously (29-33). For most continuous variables, averages of the baseline, 4-year, and 11-year values were used in regression models. For continuous variables that are linked to cardiovascular outcomes and are known to be influenced by physical activity (high-density lipoprotein cholesterol level, low-density lipoprotein [LDL] cholesterol level, body mass index [BMI], and cardiorespiratory fitness), only baseline values were used to avoid overadjusting for occupational physical activity measured during follow-up. There may still be some overadjustment because baseline values partly reflect past occupational exposures that are often highly correlated to current exposures. For cholesterol and blood pressure medications, the analyses used the proportion of examinations when medication use was reported.

Participants were considered to have IHD if they had a history of myocardial infarction or angina pectoris, currently used antiangina medication, or had positive findings of angina from the London School of Hygiene cardiovascular questionnaire (34). Baseline IMT recordings were classified by 1 physician, blind to other measures, as no atherosclerotic lesion, intima-media thickening, nonstenotic plaque, or CAS (21). Participants were not informed of ultrasound results, except for a small number who required medical attention.

Statistical analyses are described in the Appendix.

## Results

### Characteristics of the study sample

Compared to men without stenosis at baseline, men with stenosis were older, earned less money, had higher levels of LDL cholesterol and fibrinogen, had lower levels of cardiorespiratory fitness, and were more likely to take lipid-lowering or antihypertensive medication (Table 1). On average, they worked approximately 1.5 hours more per week at baseline and had longer periods of unemployment or retirement during follow-up. Similar differences were found between men with and without IHD at baseline (Table 2). Men with IHD also had lower systolic blood pressure and reported higher levels of mental strain at work than did men without IHD.

### Progression of atherosclerosis

Maximum IMT at baseline averaged 0.91 mm (standard deviation [SD], 0.21 mm; range, 0.54-2.62 mm). The average change in maximum IMT was 0.027 mm per year (SD, 0.017 mm; range, −0.033 to 0.095 mm) corresponding to a change of 0.33 mm (SD, 0.24 mm; range, −0.82 to 1.75 mm) during the 11-year follow-up. We focus on percentage change in maximum IMT, which was on average 2.72% per year (95% confidence interval [CI], 2.59%-2.85%) and 30.3% (95% CI, 28.8%-31.7%) during the entire follow-up period (average 11.13 years; SD, 0.55 years; range, 9.23-13.82 years).

### Distribution of work time

At baseline, participants worked from 3 to 7 days per week and from 16 to 91 hours per week. On average, men were employed 68% of the follow-up time and worked 1,340 hours per year (range, 23-3,922 hours), which is 71% (range, 1%-213%) of the standard Finnish work year of 1,840 hours (40 hours per week, 46 weeks per year). At each measurement and across measures of work time, 16% to 34% of participants exceeded the Finnish work time standards (Table 3).

### Measures of association between work time and progression of atherosclerosis

The relative change ratio (RCR) in maximum IMT during the 11-year follow-up period was significantly and positively associated with the number of days worked per week at baseline and annual work hours during 11 years of follow-up (Table 4). No significant associations were found with daily work hours or employment intensity. The effects varied little with incremental adjustment for covariates.

Men who worked on average 3 days per week (minimum) experienced a 23% increase in IMT, those who worked 5 days per week (median) experienced a 31% increase, and those who worked 7 days per week (maximum) experienced a 40% increase. The highest percentage change in IMT during 11 years was observed for maximum hours worked per year (Table 5).

### Percentage change in IMT by baseline cardiovascular health status

Among men with median or higher work time, men with IHD experienced consistently higher rates of IMT change than men without IHD at baseline across all exposure measures (Table 6). Significant interactions (*P* ≤ .10) were found between IHD and days per week, hours per day, and hours per week. The overall association between change in IMT and work time was mostly due to changes among the subgroup of men with IHD, and associations were significant for most measures despite the small sample size. In men without baseline IHD, marginally significant effects were observed for days worked per week and average annual work hours.

Among men with median or higher work time, men with CAS consistently experienced substantially higher rates of IMT change than men without CAS across all exposure measures (Table 7). Significant interactions (*P* ≤ .10) were found between CAS and hours per day at baseline, employment intensity, average annual work hours, and weekly work hours. In men with CAS, significant associations were observed for hours per day, week, and year; associations with days per week and employment intensity were marginally significant. In men without CAS, only the association with days per week was significant.

### Established cardiovascular risk factors, other covariates, and change in IMT

The predictive role of established cardiovascular risk factors and other covariates in the progression of atherosclerosis was determined by using the same fully adjusted regression model used in Tables 4-7. All 21 covariates were examined simultaneously in the same model that included average hours worked per year employed. Significant associations with yearly change of IMT were observed for age, baseline IMT, participation in the placebo group of an unrelated trial of lipid-lowering medication, LDL cholesterol, systolic blood pressure, proportion of follow-up time while taking lipid-lowering medication, and current regular smoking. The remaining 14 covariates including known predictors of CVD such as income, BMI, conditioning leisure-time physical activity, job stress, and plasma fibrinogen were not significant. Regression coefficients and *P* values are shown in Table 8.

## Discussion

Working 7 consecutive days per week was associated with the highest RCR of IMT, followed by the average number of hours worked per year employed, which indicates that the lack of free weekends and a high total average yearly work time are significant predictors of the progression of atherosclerosis. Weekly work hours were also positively associated with IMT change, albeit not as strongly, and were significant only in men with preexisting CVD. Hours worked per day at baseline and employment intensity during follow-up did not predict IMT change in the full cohort. However, when the subgroup of men with IHD at baseline was analyzed, hours per day, days per week, and hours per week at baseline emerged as the strongest predictors of progression of atherosclerosis, which indicates that insufficient daily and weekly rest periods pose a risk for men with IHD. In addition, significant interactions between work time and CAS were observed for all work time measures, except days per week, which had similar strong associations with IMT in all subgroups. In men with CAS, all work time measures were positively associated with progression of atherosclerosis, rendering these men especially vulnerable to the effects of long work time, as predicted by hemodynamic theory (36,37).

### Strengths and limitations

This is the first study of the effect of work time on progression of atherosclerosis, and it addresses several methodologic issues that have been identified as reasons for inconsistent findings in the literature (15). By using change in IMT as the outcome measure instead of CVD symptoms or clinical events, we circumvented the so-called healthy worker effect. Ultrasound measurements in asymptomatic populations allow an examination of the relationship between work characteristics and atherosclerosis before most disease-based selection effects occur (21,38).

The comprehensive adjustment for 21 cardiovascular risk factors is another strength of this study. The control for mental strain at work is of particular importance because associations have been reported between long working hours and perceived job stress (39,40), a factor that has also been associated with IMT change (41) and other CVD outcomes (42-44). In fact, the lack of control for job stress and other psychosocial factors has been noted by recent reviewers of the literature as a possible reason for inconsistent findings (15). The observed associations in our study are independent of stress from work deadlines and an index of 10 other self-reported mental strains at work. However, other psychosocial stressors could still contribute to the observed relationships; their potential mediating or independent roles need to be examined in future research. Work time may also be correlated with other dimensions of work that are associated with progression of atherosclerosis, including upright work posture (36,45), energy expenditure (46), and relative aerobic strain (46). However, these factors are necessarily highly correlated with work time and need to be considered possible pathways rather than confounders of the association between work time and IMT change.

To our knowledge, this is also the first prospective study of work time and cardiovascular health outcomes that analyzed both baseline and repeat work time measures. The measure of average annual hours that uses information from repeat exposure assessment is a stronger predictor of IMT change than is the corresponding baseline measure of hours per week by itself; in fact, only the former produced significant associations when the entire cohort was analyzed. However, for men with IHD, average annual work time is the weaker predictor, probably because of disease-related reduction in work hours or time off in the years after baseline. Men with IHD worked on average 358 fewer hours per year of employment during follow-up, despite working approximately 1 hour per week more at baseline than did men without IHD. This study demonstrates that analyses combining baseline and repeat exposure measures and taking into account interactions with cardiovascular health status can disentangle seemingly inconsistent results that have been characteristic in this area of research. Some highly correlated measures, such as hours per day and hours per week (correlation, 0.89) and employment intensity and average annual work hours (correlation, 0.86) differed markedly in their pattern of association and predictive strengths. The different measures may reflect different work-rest patterns, and the issue of adequate rest periods needs to be explored further in future research.

A limitation of this study is that work time measures were based on self-report (with the exception of duration of retirement, which was assessed by administrative records). However, a random sample of 54-year-olds from our study showed good 12-month test-retest reliability (interclass correlation coefficient, 0.69) for a summary measure of occupational activities evaluated in our interview instrument (27). Other investigators have noted that self-reported work hours are the most reliable item in occupational activity questionnaires (eg, 2-week test-retest intraclass correlation coefficient of 0.91) (47).

Despite high instrument reliability and extensive data checking, extreme values of work time could inflate reported RCRs because RCRs were based on the ratio between maximum and minimum values of relative change for each work time measure. However, maximum and minimum values reported in this study were not considered outliers because they reflect the actual experience of many study participants and of many working adults in the general population (48,49).

### Biological plausibility and potential causal pathways

If a work environment is unhealthy, then working more hours will naturally be more unhealthy. Potential occupational risk factors for CVD include physical (eg, noise), chemical (eg, carbon disulfide, carbon monoxide, diesel exhaust), and psychosocial (eg, job strain, effort-reward imbalance) stressors (42,50-54). Work hours may also affect health-related behavior, such as smoking, drinking alcohol, diet, and exercise (13,55,56). In addition, long work time may impair work-life balance and erode social support and recovery off work (57). Given the variety of 100 occupations in this population-based sample, more than 1 pathway can be expected to mediate the observed effects of long work times. We found little variation of effects after adjusting for behavioral risk factors, which suggests that the effect of work time on IMT progression is not mediated in any substantial way by alcohol consumption, smoking, leisure-time physical activity, or cardiorespiratory fitness. Similarly, income and psychosocial job factors do not appear to affect the association between work hours and IMT progression.

Our findings of accelerated progression of atherosclerosis in men with preexisting CVD are consistent with the hemodynamic theory of atherosclerosis (37). Building on this theory, we propose that mental arousal, emotional reactivity, and physical activities on the job are likely to increase average heart rate, which leads to changes in blood flow that cause arterial wall injury and inflammation and, ultimately, atherosclerosis (36). An increased heart rate shortens the cumulative time spent in diastole, the heart's relaxation phase, when wall shear stress is optimal, and leads to more time spent in systole, the heart's contraction phase, when wall shear stress fluctuates in a suboptimal range and intravascular turbulence increases (37). Increased turbulence and the resulting reduction in shear stress at the arterial walls are considered to be primary causes of endothelial damage in human arteries (37,58,59). This damage sets the stage for lipids and other pathogenic substances to be absorbed into arterial walls, which leads to the inflammatory process that is believed to be the basis of intima-media thickening, the formation of atherosclerotic plaques, and eventual stenosis of the arteries (60). Progression of lumen-reducing stenoses in turn leads to post-stenotic vessel dilatation and suboptimal post-stenotic wall shear stress because wall shear stress is an exponential function of vessel radius. These hemodynamic mechanisms can explain the increased rates of change in IMT observed among men with baseline CAS. They also explain the higher rates of progression of atherosclerosis associated with a standing work posture among people with preexisting CAS than among those without CAS (36) and the observed positive associations between energy expenditure at work and 11-year progression of carotid atherosclerosis in the same sample (46).

The observed associations between work time and IMT change may be mediated by some dimensions of occupational physical activity or other characteristics of the work environment that were not investigated in this report. We focused on work time itself and how different measures of work time predict progression of atherosclerosis. Ascertaining the specific role of some of these more proximal occupational factors is beyond the scope of this report. Future research needs to clarify the specific mechanisms and biological and occupational pathways involved. However, the observed patterns of association in this study and the interaction effects with IHD and CAS have implications for CVD prevention regardless of the specific mechanisms involved.

### Implications for disease prevention and needs for future research

Aging workers, especially those with preexisting CVD, could benefit from reducing their work time and should be made aware of this possibility in general, cardiologic, occupational, and rehabilitation medical practice. From a public health perspective, population-based approaches to reduce excessive work time may help prevent the progression of arteriosclerosis and associated CVD. Work time reduction may also help workers maintain their ability to work despite age-related declines in cardiorespiratory fitness and increases in the prevalence of degenerative musculoskeletal diseases (61). The European Union recently enacted legislation regulating maximum work time and minimum rest periods covering more than 495 million people. The current European Working Time Directive and respective Canadian regulations still allow regular 48-hour workweeks and fail to recognize the special needs of aging workers. In our study, atherogenic effects were already visible at median values of work intensity measures (ie, working traditional regular schedules of 5 days per week or 40 hours per week). Current regulations may not provide sufficient protection, especially for middle-aged workers with preexisting IHD or CAS.

Furthermore, the lack of work-time regulation in some countries needs to be seen as a potential public health hazard, especially when combined with a high prevalence of overtime work (such as in Australia, Japan, and the United States). Income inequality, a factor that has been linked to higher rates of CVD (62,63), may prompt large numbers of low-income workers to hold more than 1 job, which extends work time to extremes. In Japan and most European Union countries, annual working hours have decreased substantially during the last economic cycle before 1994, while hours increased substantially in the United States and United Kingdom (64). The effect of the new European Working Time Directive on UK work hours is not yet known, but the absence of any work-time regulation in the United States, combined with increasing income inequality and the corresponding large low-wage sector, may increase CVD risk for many working Americans.

This investigation used some readily available and easily reproducible work-rest ratios and examined typical overall work-time arrangements prevalent during the study period. Specific shift schedules and other emerging flexible patterns of work such as contingent work, compressed work weeks, and averaged work weeks need to be addressed in future research. Future studies also need to determine age-, sex-, and occupation-specific relationships and attributable risks associated with specific work-time schedules to facilitate workplace and public health prevention efforts. Additional research is also needed to determine the effects of long work hours on musculoskeletal and other disease outcomes, employment, productivity, work performance, safety, motivation, absenteeism, job turnover, and work-life balance to fully understand the overall costs and benefits of long working hours (64).

Drawing causal inferences from our data is difficult because of the multiple possible pathways involved. However, the case for causal inference is strengthened by the biological plausibility of several pathways, consistency with extant knowledge about cardiovascular risk factors, and the methodologic strength of the study, including the use of repeat exposure measures (reducing misclassification bias), objective subclinical outcome measures (reducing selection bias), comprehensive control for confounding, and the prospective study design that excludes the possibility of reverse causation.

### Conclusions

Approximately one-third of middle-aged men work more than the standard 40-hour work week. Work time is positively related to accelerated progression of carotid atherosclerosis. Men with preexisting IHD or CAS appear to be especially vulnerable to the effects of long work times. Findings are consistent with the hemodynamic theory of atherosclerosis and known occupational risk factors for CVD. Regardless of the specific occupational conditions that may constitute the pathways for the observed relationships, findings suggest that reducing weekly and yearly work time could have cardiovascular and public health benefits, especially in the aging working population.

## Figures and Tables

**Table 1:** Characteristics of the Study Sample and Distribution of Independent Variables by Carotid Artery Stenosis Status at Baseline, Kuopio Ischemic Heart Disease Risk Factor Study, 1986-2001 (N = 621)

**Variable**	Men Without Stenosis (n = 492)	Men With Stenosis (n = 129)	*P* Value^a^
**Age and technical factors**			
Mean (SD) age at baseline, y	48.4 (5.6)	53.7 (5.0)	.001
Mean (SD) log of maximum IMT at baseline, log mm	−0.036 (0.178)	0.138 (0.230)	.001
Sonographer at 11-year follow-up,^b^ n (%)			
A	16 (3.3)	5 (3.9)	.193
B	3 (0.6)	3 (2.3)	
C	473 (96.1)	121 (93.8)	
Participation in lipid-lowering drug trial, n (%)			
Placebo group	33 (6.7)	10 (7.8)	.677
Treatment group	29 (5.9)	11 (8.5)	.278
**Biological factors**			
Mean (SD) blood glucose level, mmol/L^c^	4.9 (0.8)	5.0 (0.8)	.445
Mean (SD) plasma fibrinogen level, g/L^c^	3.0 (0.5)	3.2 (0.4)	.001
Mean (SD) BMI at baseline, kg/m^2^	26.6 (3.2)	26.4 (3.2)	.579
Mean (SD) LDL cholesterol at baseline, mmol/L	3.8 (0.9)	4.0 (0.9)	.004
Mean (SD) HDL cholesterol at baseline, mmol/L	1.3 (0.3)	1.3 (0.3)	.535
Mean (SD) SBP at baseline, mm Hg	130.5 (14.4)	132.4 (15.6)	.185
Mean (SD) proportion of follow-up time taking lipid-lowering medication	0.01 (0.06)	0.04 (0.13)	.034
Mean (SD) proportion of follow-up time taking blood pressure-lowering medication	0.16 (0.31)	0.30 (0.40)	.001
**Behavioral factors**			
Mean (SD) alcohol consumption, g/wk^c^	79.6 (96.9)	72.9 (104.1)	.488
Smoking status, n (%)			
Nonsmoker	199 (40.5)	33 (25.6)	.007
Former smoker	136 (27.6)	44 (34.1)	
Irregular smoker	40 (8.3)	8 (6.2)	
Current smoker	117 (23.8)	44 (34.1)	
Mean (SD) conditioning LTPA, h/y^c^	118.7 (100.3)	128.1 (106.5)	.353
Mean (SD) cardiorespiratory fitness, mL O_2_/kg/min	34.4 (7.1)	30.2 (6.9)	.001
**Socioeconomic status**			
Mean (SD) annual income, 1,000 FIM^c^	12.2 (6.9)	9.7 (5.0)	.001
**Psychosocial job factors**			
Mean (SD) social support at work score	5.9 (2.2)	6.0 (2.6)	.775
Mean (SD) mental strain at work index	12.0 (5.1)	12.2 (6.0)	.670
Stress from work deadlines, n (%)	174 (35.4)	50 (38.8)	.475
**Work time**			
Mean (SD) days/week at baseline	5.2 (0.6)	5.3 (0.8)	.161
Mean (SD) hours/day at baseline	8.0 (1.5)	8.1 (1.6)	.438
Mean (SD) hours/week at baseline	41.9 (11.3)	43.5 (13.2)	.201
Employment intensity between baseline and 11-year follow-up	0.73 (0.31)	0.50 (0.31)	.001
Mean (SD) hours worked per year employed between baseline and 11-year follow-up	1,384 (697)	1,006 (719)	.001

Abbreviations: SD, standard deviation; IMT, intima-media thickness; BMI, body mass index; LDL, low-density lipoprotein; HDL, high-density lipoprotein; SBP, systolic blood pressure; LTPA, leisure-time physical activity; FIM, Finnish markka.

a Calculated by using *t* tests or χ^2^ tests.

b All ultrasound examinations were performed by the same sonographer at baseline.

c Average of baseline, 4-year, and 11-year values.

**Table 2:** Characteristics of the Study Sample and Distribution of Independent Variables by IHD Status at Baseline, Kuopio Ischemic Heart Disease Risk Factor Study, 1986-2001 (N = 621)

**Variable**	Men Without IHD (n = 542)	Men With IHD (n = 79)	*P* value^a^
**Age and technical factors**			
Mean (SD) age at baseline, y	49.1 (5.9)	52.3 (5.5)	.001
Mean (SD) log of maximum IMT at baseline, log mm	−.013 (0.19)	−.052 (0.25)	.011
Sonographer at 11-year follow-up,^b^ n (%)			
A	19 (3.5)	2 (2.5)	.577
B	6 (1.1)	0	
C	517 (95.4)	77 (97.5)	
Participation in lipid-lowering drug trial, n (%)			
Placebo group	37 (6.8)	6 (7.6)	.802
Treatment group	34 (6.3)	6 (7.6)	.655
**Biological factors**			
Mean (SD) blood glucose level, mmol/L^c^	4.9 (0.9)	4.9 (0.7)	.808
Mean (SD) plasma fibrinogen level, g/L^c^	3.0 (0.5)	3.1 (0.5)	.025
Mean (SD) BMI at baseline, kg/m^2^	26.5 (3.2)	26.8 (3.3)	.405
Mean (SD) LDL cholesterol at baseline, mmol/L	3.8 (0.9)	3.9 (1.0)	.232
Mean (SD) HDL cholesterol at baseline, mmol/L	1.3 (0.3)	1.3 (0.3)	.634
Mean (SD) SBP at baseline, mm Hg	131 (15)	127 (15)	.017
Mean (SD) proportion of follow-up time taking lipid-lowering medication	0.01 (0.07)	0.04 (0.13)	.061
Mean (SD) proportion of follow-up time taking blood pressure-lowering medication	0.17 (0.32)	0.33 (0.41)	.002
**Behavioral factors**			
Mean (SD) alcohol consumption, g/wk^c^	77.3 (996.6)	84.5 (110.1)	.543
Smoking status, n (%)			
Nonsmoker	204 (37.6)	28 (35.4)	.968
Former smoker	157 (29.0)	23 (29.1)	
Irregular smoker	41 (7.6)	7 (8.9)	
Current smoker	149 (25.8)	21 (26.6)	
Mean (SD) conditioning LTPA, h/y^c^	121 (103)	120 (93)	.986
Mean (SD) cardiorespiratory fitness, mL O_2_/kg/min	34.3 (7.0)	28.4 (6.8)	.001
**Socioeconomic status**			
Mean (SD) annual income, 1,000 FIM^c^	12.0 (6.8)	9.2 (4.5)	.001
**Psychosocial job factors**			
Mean (SD) social support at work score	6.0 (2.3)	5.7 (2.4)	.410
Mean (SD) mental strain at work index	11.8 (5.2)	13.4 (5.4)	.014
Stress from work deadlines, n (%)	351 (64.8%)	33 (58.2%)	.259
**Work time**			
Mean (SD) days/week at baseline	5.2 (0.6)	5.3 (0.8)	.161
Mean (SD) hours/day at baseline	8.0 (1.5)	8.1 (1.6)	.438
Mean (SD) hours/week at baseline	41.9 (11.3)	43.5 (13.2)	.201
Employment intensity between baseline and 11-year follow-up	0.70 (0.31)	0.51 (0.32)	.001
Mean (SD) hours worked per year employed between baseline and 11-year follow-up	1,351 (708)	993 (711)	.001

Abbreviations: IHD, ischemic heart disease; SD, standard deviation; IMT, intima-media thickness; BMI, body mass index; LDL, low-density lipoprotein; HDL, high-density lipoprotein; SBP, systolic blood pressure; LTPA, leisure-time physical activity; FIM, Finnish markka.

a Calculated by using *t* tests or χ^2^ tests.

b All ultrasound examinations were performed by the same sonographer at baseline.

c Average of baseline, 4-year, and 11-year values.

**Table 3:** Percentage of Men Who Exceeded Finnish Work Time Standards by Measure of Work Time, Kuopio Ischemic Heart Disease Risk Factor Study, 1986-2001 (N = 621)

**Work Time Measure**	% Who Exceeded Standard
**Days/week (standard: 5)**	
Baseline	18.2
4-year follow-up	16.2
11-year follow-up	20.8
**Hours/day (standard: 8)**	
Baseline	30.8
4-year follow-up	28.2
11-year follow-up	30.4
**Hours/week (standard: 40)**	
Baseline	33.3
4-year follow-up	31.3
11-year follow-up	33.6
**Average hours worked per year employed (standard: 1,840)^a^**	
From baseline to 4-year follow-up	26.6
From 4-year follow-up to 11-year follow-up	15.9
From baseline to 11-year follow-up	17.4

a Weighted average of yearly work hours per year employed during follow-up.

**Table 4:** RCR^a^ in Maximum IMT During 11-Year Follow-Up, by Measure of Work Time, Kuopio Ischemic Heart Disease Risk Factor Study, 1986-2001 (N = 621)

Work Time Measure	Model 1^b^		Model 2^c^		Model 3^d^		Model 4^e^	

	RCR (95% CI)	*P* Value	RCR (95% CI)	*P* Value	RCR (95% CI)	*P* Value	RCR (95% CI)	*P* Value
D/wk at baseline	1.12 (1.03–1.22)	.008	1.11 (1.02-1.21)	.016	1.14 (1.05-1.25)	.002	1.14 (1.04-1.24)	.003
H/d at baseline	1.02 (0.92–1.13)	.665	1.02 (0.92-1.12)	.743	1.01 (0.92-1.12)	.815	1.01 (0.92-1.12)	.781
H/wk at baseline	1.07 (0.98–1.67)	.123	1.06 (0.97-1.16)	.171	1.07 (0.98-1.17)	.105	1.07 (0.98-1.17)	.118
Employment intensity after baseline	1.00 (0.95-1.06)	.863	1.00 (0.95-1.06)	.864	1.01 (0.96-1.07)	.630	1.02 (0.96-1.08)	.501
Average hours worked per year employed after baseline	1.09 (1.00-1.19)	.041	1.07 (0.99-1.17)	.098	1.09 (1.00-1.19)	.047	1.10 (1.01-1.20)	.038

Abbreviations: RCR, relative change ratio; IMT, intima-media thickness; CI, confidence interval.

a RCR per unit change in IMT where unit is the observed range (maximum − minimum) in work time.

b Model 1: adjusted for age and technical factors (listed in Table 1).

c Model 2: model 1 with additional adjustment for biological factors (Table 1).

d Model 3: model 2 with additional adjustment for behavioral factors (Table 1).

e Model 4: model 3 with additional adjustment for socioeconomic status and psychosocial job factors (total of 21 covariates — Table 1).

**Table 5:** Percentage Change in Maximum IMT and RCR^a^ by Work Time Measure During 11-Year Follow-Up, Kuopio Ischemic Heart Disease Risk Factor Study, 1986-2001 (N = 621)^b^

**Work Time Measure**	Minimum, % (95% CI)	Median, % (95% CI)	Maximum, % (95% CI)	RCR (95% CI)	*P* Value
D/wk at baseline	23.1 (16.1-30.4)	31.3 (27.6-35.2)	40.2 (33.9-46.7)	1.14 (1.04-1.24)	.003
H/d at baseline	32.0 (25.5-38.8)	32.7 (29.0-36.5)	33.9 (25.4-43.0)	1.01 (0.92-1.12)	.781
H/wk at baseline	29.5 (24.1-35.1)	32.1 (28.3-36.0)	38.9 (30.4-47.9)	1.07 (0.98-1.17)	.118
Employment intensity after baseline	31.1 (25.0-37.4)	33.0 (29.2-36.8)	33.6 (29.3-38.2)	1.02 (0.96-1.08)	.501
Average hours worked per year employed after baseline	28.8 (23.7-34.1)	32.8 (29.1-36.6)	41.1 (32.4-50.4)	1.10 (1.01-1.20)	.038

Abbreviations: IMT, intima-media thickness; RCR, relative change ratio; CI, confidence interval.

a RCR per unit change in IMT where unit is the observed range (maximum − minimum) in work time.

b Results are from multiple regression analyses that adjusted for all 21 covariates listed in Table 1.

**Table 6:** Eleven-Year Percentage Change in Maximum IMT at Minimum, Median, and Maximum Levels of Work Time; Measures of Association Between Work Time and IMT Progression (RCR)^a^; and Interactions of Work Time With Baseline IHD, Kuopio Ischemic Heart Disease Risk Factor Study, 1986-2001 (N = 621)^b^

Work Time Measure	Men Without IHD at Baseline (n = 542)				

	Minimum, % (95% CI)	Median, % (95% CI)	Maximum, % (95% CI)	RCR (95% CI)	*P* Value
Days/week at baseline	24.8 (17.4-32.8)	30.7 (27.0-34.6)	36.9 (30.3-43.8)	1.10 (1.00-1.21)	.057
Hours/day at baseline	33.5 (26.6-40.7)	32.0 (28.3-35.9)	29.5 (20.8-38.8)	0.97 (0.87-1.08)	.580
Hours/week at baseline	30.6 (24.9-36.5)	31.5 (27.7-35.4)	33.7 (25.1-43.0)	1.02 (0.93-1.13)	.620
Employment intensity after baseline	28.9 (22.7-35.4)	32.2 (28.4-36.0)	33.4 (28.9-38.0)	1.03 (0.98-1.10)	.259
Average hours worked per year employed after baseline	28.2 (22.9-33.7)	31.9 (28.2-35.8)	39.7 (30.6-49.4)	1.09 (0.99-1.20)	.066

Table 6. (continued)							
Work Time Measure	Men With IHD at Baseline (n = 79)				*P* Value	RCR Ratio (RCR_IHD_/RCR_no IHD_)	*P* Value for Interaction

	Minimum, % (95% CI)	Median, % (95% CI)	Maximum, % (95% CI)	RCR (95% CI)			
Days/week at baseline	16.9 (3.7-31.8)	35.6 (29.3-42.4)	57.4 (43.2-73.1)	1.35 (1.11-1.63)	.003	1.23 (0.99-1.52)	.058
Hours/day at baseline	20.9 (6.9-36.7)	36.5 (30.0-43.2)	68.9 (42.6-100.0)	1.40 (1.06-1.84)	.018	1.44 (1.07-1.94)	.016
Hours/week at baseline	22.6 (11.8-34.4)	35.3 (28.9-42.1)	73.1 (49.1-101.0)	1.41 (1.13-1.76)	.002	1.38 (1.09-1.75)	.008
Employment intensity after baseline	41.3 (30.5-53.1)	36.9 (29.9-44.4)	36.9 (26.0-45.6)	0.96 (0.85-1.08)	.480	0.95 (0.87-1.03)	.225
Average hours worked per year employed after baseline	32.8 (23.6-42.7)	39.6 (32.8-46.6)	53.8 (31.0-81.0)	1.16 (0.94-1.43)	.166	1.06 (0.85-1.33)	.589

Abbreviations: IMT, intima-media thickness; RCR, relative change ratio; IHD, ischemic heart disease; CI, confidence interval.

a RCR per unit change in IMT where unit is the observed range (maximum − minimum) in work time.

b Results are from multiple regression analyses that adjusted for all 21 covariates listed in Table 1.

**Table 7:** Eleven-Year Percentage Change in Maximum IMT at Minimum, Median, and Maximum Levels of Work Time; Measures of Association Between Work Time and IMT Progression (RCR)^a^; and Interactions of Work Time With Baseline CAS, Kuopio Ischemic Heart Disease Risk Factor Study, 1986-2001 (N = 621 Men)^b^

Work Time Measure	Men Without CAS at Baseline (n = 492)				

	Minimum, % (95% CI)	Median, % (95% CI)	Maximum, % (95% CI)	RCR (95% CI)	*P* Value
Days/week at baseline	23.1 (15.2-31.5)	30.9 (27.1-34.9)	39.3 (32.3-46.7)	1.13 (1.02-1.26)	.019
Hours/day at baseline	34.9 (27.6-42.5)	32.6 (28.9-36.5)	28.8 (19.7-38.6)	0.96 (0.85-1.07)	.435
Hours/week at baseline	31.1 (25.2-37.4)	32.1 (28.2-36.0)	34.4 (25.2-44.3)	1.03 (0.93-1.14)	.635
Employment intensity after baseline	32.7 (26.1-39.7)	32.4 (28.7-36.3)	32.3 (27.9-36.9)	1.00 (0.94-1.06)	.917
Average hours worked per year employed after baseline	30.6 (25.1-36.4)	32.4 (28.8-36.2)	36.1 (26.9-45.9)	1.04 (0.95-1.15)	.405

Table 7. (continued)							
Work Time Measure	Men With CAS at Baseline (n = 129)					RCR Ratio (RCR_CAS_/RCR_no CAS_	*P* Value for Interaction

	Minimum, % (95% CI)	Median, % (95% CI)	Maximum, % (95% CI)	RCR (95% CI)	*P* Value
D/wk at baseline	26.1 (14.5-39.0)	35.0 (29.1-41.2)	44.5 (33.8-56.0)	1.15 (0.99-1.33)	.078	1.01 (0.85-1.21)	.897
H/d at baseline	25.5 (14.0-38.1)	35.4 (29.5 41.5)	54.6 (35.8-76.0)	1.23 (1.00-1.52)	.049	1.29 (1.02-1.64)	.036
H/wk at baseline	27.7 (18.5-37.6)	34.8 (28.9-41.0)	54.3 (37.4-73.3)	1.21 (1.02-1.43)	.028	1.18 (0.97-1.43)	.100
Employment intensity after baseline	30.6 (21.9-39.8)	39.5 (33.0-46.4)	42.8 (34.1-52.0)	1.09 (0.99-1.20)	.070	1.10 (0.99-1.22)	.082
Average hours worked per year employed after baseline	27.9 (20.2-36.0)	38.7 (32.6-45.0)	62.5 (43.5-84.1)	1.27 (1.08-1.49)	.003	1.22 (1.02-1.46)	.030

Abbreviations: IMT, intima-media thickness; RCR, relative change ratio; CAS, coronary artery stenosis; CI, confidence interval.

a RCR per unit change in IMT where unit is the observed range (maximum − minimum) in work time.

b Results are from multiple regression analyses that adjusted for all 21 covariates listed in Table 1

**Table 8:** Associations of Covariates With Yearly Change in Ln-Transformed Maximum IMT in Multivariate^a^ Regression Analyses, Kuopio Ischemic Heart Disease Risk Factor Study, 1986-2001 (N = 621 Men)

Variable	Multiple Regression Adjusted for All Covariates^a^	

	Coefficient	*P* Value
**Work time:** average hours worked per year employed after baseline	.00214	.038
**Age and technical factors**		
Age at baseline, y	.000506	.001
Log of maximum IMT at baseline, log mm	−.035111	.001
Sonographer at 11-year follow-up^b^		
A	Reference	
B	−.000717	.834
C	.001979	.761
Participation in lipid-lowering drug trial		
Placebo group	−.005981	.024
Treatment group	−.005172	.054
**Biological factors**		
Mean blood glucose level, mmol/L^c^	.000385	.620
Mean plasma fibrinogen level, g/L^c^	.000466	.754
BMI at baseline, kg/m^2^	.000358	.121
LDL cholesterol at baseline, mmol/L	.002739	.001
HDL cholesterol at baseline, mmol/L	.000667	.774
SBP at baseline, mm Hg	.000099	.035
Proportion of follow-up time taking lipid-lowering medication	−.017354	.035
Proportion of follow-up time taking blood pressure-lowering medication	−.000299	.887
**Behavioral factors**		
Alcohol consumption average, g/wk^c^	−.000003	.679
Smoking status		
Nonsmoker	Reference	
Former smoker	.001566	.315
Irregular smoker	.002313	.356
Current smoker	.007601	.001
Mean conditioning LTPA (hours/year)^c^	−.000006	.351
Cardiorespiratory fitness (mL O_2_/kg/min)	−.000059	.584
**Socioeconomic status**		
Mean annual income, FIM^c^	−.000159	.101
**Psychosocial job factors**		
Mean social support at work score^c^	−.000099	.715
Mean mental strain at work index^c^	−.000114	.369
Stress from work deadlines^d^	−.000047	.973
Model constant	.022928	.001

Abbreviations: IMT, intima-media thickness; BMI, body mass index; LDL, low-density lipoprotein; HDL, high-density lipoprotein; SBP, systolic blood pressure; LTPA, leisure-time physical activity; FIM, Finnish markka.

a Multiple linear regression with average hours worked per year employed and all covariates listed in this table entered simultaneously into 1 model.

b All ultrasound examinations were performed by the same sonographer at baseline.

c Average of baseline, 4-year, and 11-year values.

d Combination of baseline and 4-year follow-up; not assessed at 11-year follow-up.

## Appendix. Statistical Analysis

The baseline characteristics of men with and without cardiovascular disease (ischemic heart disease [IHD] or coronary artery stenosis [CAS]) were compared by using *t* tests for continuous variables and χ^2^ tests for categorical variables. To study the progression of maximal intima-media thickness (IMT), we used multiple linear regression analysis in Stata version 9.2 (StataCorp LP, College Station, Texas). The outcome for these analyses was [ln(*y_F_*)−ln(*y_I_*)]/Δ*t*, where *y_I_* is the initial maximum IMT at baseline and *y_F_* is the final maximum IMT at the follow-up examination Δ*t* years after the baseline examination. The maximum IMT values at baseline and follow-up were ln-transformed because doing so normalized the original skewed maximum IMT measurements. Also, the residual distribution of the changes in ln(maximum IMT) was more nearly normal than changes based on maximum IMT without transformation. The division by Δ*t* handles variation from the nominal follow-up time of 11 years by expressing change on a per-year basis. In these analyses, we included a predictor based on a measure of work-time intensity along with some or all of the 21 covariates listed in Table 1. Continuous covariates were centered at the mean.

The use of changes in ln-transformed maximum IMT leads naturally to interpretation of results in terms of relative change and percentage change. Relative change is calculated as *RC* = *y_F_*/*y_I_*, and [ln(*y_F_*) − ln(*y_I_*)]/Δ*t =* ln(*y_F_*/*y_I_*)/Δ*t =* ln(*RC*)/Δ*t*. Consequently, for any specified values of predictors, the fitted model provides a way to estimate average ln(*RC*)/Δ*t*, symbolized as *E*[ln(*RC*)]/Δ*t*. A corresponding estimate of *E*(*RC*) over *K* years instead of per year is obtained from *E*[*RC*]_*K*_ = Φexp(*E*[ln(*RC*)]*K*/Δ*t*), where Φ is the back-transformation correction factor (35), which, with our data, was so close to 1 that it had no effect. Correspondingly, the average percentage change for *K* years is *E*[*RC*]_*K*_ = 100(*E*[*RC*]_*K*_ − 1).

Several tables present the estimated expected average percentage change for 11 years with the coefficients from the fitted model. We calculated estimated relative change for the minimum, median, and maximum value for each work-time measure. Other variables were set to 0, which corresponds to using the mean value for centered continuous variables and the reference level coded 0 for any predictors used to represent categorical variables. We also studied whether or not the association between the work-time variable and the outcome was different for the subgroups with and without IHD at baseline. Similar subgroup-specific results were examined for subgroups with and without CAS at baseline.

The relative change ratio (RCR), defined as the ratio of the relative change at a comparison level of a predictor of interest divided by the relative change at a reference level for the predictor, provides a summary measure of association between a work-time measure, *x*_1_, and the outcome. The RCR depends on years of follow-up (*K*). With a multiple regression model, *E*[ln(*RC*)/Δ*t*] = *B*_0_ + *B*_1_ *x*_1_ + … + *B_P_x_P_*, in which no interaction terms involve the predictor, *x*_1_, the RCR for *K* years of follow-up is exp(*B*_1_Δ_1_ *K*) where Δ_1_ = *x*_1_^C^ − *x*_1_^R^ is the difference between the comparison level and the reference level for the predictor *x*_1_.

To check the adequacy of a simple linear representation of the work time variables, we assessed whether significantly improved fit resulted from using both linear and quadratic terms in the fully adjusted model. Models without the quadratic term were not rejected in favor of those with the quadratic terms. However, because our outcome measure was log-transformed, all relationships found in these linear models represent a curvilinear exponential dose-response relationship.
